# Effects of renal denervation on cardiac oxidative stress and local activity of the sympathetic nervous system and renin-angiotensin system in acute myocardial infracted dogs

**DOI:** 10.1186/s12872-017-0498-1

**Published:** 2017-02-17

**Authors:** Qiaoli Feng, Chengzhi Lu, Li Wang, Lijun Song, Chao Li, Ravi Chandra Uppada

**Affiliations:** 1 First Center Clinic College of Tianjin Medical University, Tianjin First Central Hospital, Tianjin, China; 2 Department of Cardiology, Tianjin First Central Hospital, 24 Fukang Road, Nankai District, Tianjin, 300192 China; 3 Department of Digestion, Tianjin First Central Hospital, Tianjin, China

**Keywords:** Renal denervation, Acute myocardial infarction, Oxidative stress, Sympathetic nervous system, Renin-angiotensin system

## Abstract

**Background:**

This study sought to evaluate the therapeutic effects of renal denervation (RDN) on acute myocardial infarction (MI) in canines and explore its possible mechanisms of action.

**Methods:**

Eighteen healthy mongrel dogs were randomly assigned to either the control group, the MI group or the MI + RDN group. To assess cardiac function, left ventricular ejection fraction (LVEF), left ventricular end-diastolic dimension (LVEDD), left ventricular end-systolic dimension (LVESD) and fraction shortening (FS) were recorded. Additionally, haemodynamic parameters such as left ventricular systolic pressure (LVSP), left ventricular end-diastolic pressure (LVEDP) and heart rate (HR) were measured. Cardiac oxidative stress levels were evaluated based on the expression of p47^phox^ mRNA, malondialdehyde (MDA), anti-superoxide anion free radical (ASAFR) and activity of superoxide dismutase (SOD). To measure the local activity of the sympathetic nervous system (SNS) and renin-angiotensin system (RAS), the levels of tyrosine hydroxylase (TH), angiotensin II (AngII), angiotensin-converting enzyme 2 (ACE2), angiotensin (1–7) [Ang(1–7)] and Mas receptor (MasR) in myocardial tissues were recorded. The expression of TH in renal tissue and serum creatinine were used to assess the effectiveness of the RDN procedure and renal function, respectively.

**Results:**

We found that MI deteriorated heart function and activated cardiac oxidative stress and the local neurohumoral system, while RDN partially reversed these changes. Compared with the control group, parameters including LVEDD, LVESD, LVEDP and the levels of ASAFR, MDA, p47^phox^,ACE2, Ang(1–7), MasR, AngII and TH-positive nerves were increased (all *P* < 0.05) in myocardial infracted dogs; meanwhile, LVEF, FS, LVSP and SOD expression were decreased (all *P* < 0.05). However, after RDN therapy, these changes were significantly improved (*P* < 0.05), except that there were no significant differences observed in FS or LVSP between the two groups (*P* = 0.092 and 0.931, respectively). Importantly, the expression of TH, AngII and Ang(1–7) was positively correlated with MDA and negatively correlated with SOD. Between-group comparisons demonstrated no differences in serum creatinine (*P* = 0.706).

**Conclusions:**

RDN attenuated cardiac remodelling and improved heart function by decreasing the level of cardiac oxidative stress and the local activity of the SNS and RAS in cardiac tissues. Additionally, the safety of the RDN procedure was established, as no significant decrease in LVSP or rise in serum creatinine was observed in our study.

## Background

The incidence of acute myocardial infarction (MI) has increased over time. Chronic heart failure (CHF) is a common complication of acute MI, which places substantial burden on the health care system. Complicated pathophysiologic mechanisms participate in the development of HF, among which the whole-body and local cardiac sympathetic nervous system (SNS) and renin-angiotensin system (RAS) contribute significantly to the pathogenesis of CHF. Recently, oxidative stress has been demonstrated to play a pivotal role in ventricular remodelling after MI. In the clinical setting, drugs targeting these two systems, such as angiotensin-converting enzyme inhibitors(ACEI), angiotensin receptor blockers(ARB), mineralocorticoid receptor antagonists, and beta-blockers, have been the mainstay of management for post-MI HF. Additionally, anti-oxidative therapy has attracted increasing attention in the cardiovascular field. However, despite intensive therapeutic treatment, a large number of patients continue to experience worsening of HF symptoms. Thus, it remains necessary to explore new therapeutic approaches for the treatment of MI and its complications.

Previously published studies have shown that renal denervation (RDN) provides significant benefits in delaying the progression of cardiac hypertrophy [1], attenuating left ventricular (LV) remodelling and improving cardiac function [2]. The activities of the systemic SNS and RAS [3, 4] were shown to be decreased by RDN through interruption of the renal sympathetic efferent and afferent nerves [5]. However, the effects of RDN on cardiac-specific SNS and RAS activity and oxidative stress have not been investigated. In the present study, we used an established canine MI model to explore the related mechanisms, and thus further validate that RDN could be used as a new treatment for post-MI remodelling and HF.

## Methods

### Animals

Eighteen healthy mongrel dogs (male:female, 8:10), weighing 15 ~ 18 kg, were used in this study (and were provided by the Experimental Animal Centre in Tianjin). The experiments were carried out in strict accordance with the guide for the care and use of laboratory animals. The animals were kept under a 12/12-h light/dark cycle and fed with regular chow and water ad libitum.

### Experimental groups and conditions

All experimental mongrel canines were randomly divided into three groups. (1) The control group (*n* = 6) received only a coronary angiogram, followed by a renal arteriogram one week later. (2) The MI group (*n* = 6) underwent an established procedure inducing MI and, one week later, underwent a renal arteriogram. (3) The MI + RDN group (*n* = 6) underwent the MI-inducing procedure, followed by renal denervation one week later.

### Myocardial infarction

All animals were anesthetized with 6% sodium pentobarbital (30 mg/kg, intravenous (IV)), with an additional 50 mg given as needed through IV injection every 30 to 60 min, according to the reaction of the animals. The dogs were placed on the operating table in a supine position. Using a Mac-Lab (GE, United States) hemodynamic detection system, electrocardiography (ECG) and vital signs were monitored. The right iliac region was shaved and disinfected, and then a venous pathway was established. The right femoral artery was punctured, and then a 6 F guiding wire was inserted through the sheath, followed by the injection of 1000 IU heparin. After verifying the coronary anatomy, a bolus of gelatine sponge (1 mm in diameter) was injected into the distal end of the fist diagonal branch of the left anterior descending artery, as described previously [6]. Coronary angiography was performed again 10 min later to confirm interruption of the blood flow in the distal vessels. The operation lasted 60–90 min.

### Renal denervation

Under pentobarbital sodium (30 mg/kg, IV) anaesthesia, bilateral renal denervation was performed in the MI + RDN group; meanwhile, in the control and MI groups, the operation was the same except that the nerves were kept intact. The right femoral groin was shaved prior to connecting the radio frequency ablation apparatus (IBI-1500 T, IBI, United States). The temperature of the radiofrequency ablation instrument was set at 43 °C, at a power of 10 W, for a duration of 90 s. The right femoral artery was punctured, and a 6 F guiding wire was inserted through the guiding sheath. Renal angiography was performed to determine the location of the renal artery. The ablation electrode (6 F ablation catheter tip, electrode length of 4 mm) was then inserted, and radio frequency (RF) energy was applied to the endothelial lining. The catheter tip was placed in the proximal portion of the renal artery main trunk, and the ablation procedure was conducted by applying RF energy in the renal artery distally to proximally and circumferentially. Then, the catheter was withdrawn 1–2 cm to generate another ablation location. Renal angiography was performed again to validate that the catheter tip was well-attached to the vessel wall before ablating at the new target site. This procedure was repeated three to four times in the renal artery, and then the same RF energy was applied to the contralateral renal artery.

### Transthoracic echocardiography

To evaluate LV function and cardiac chamber structure, echocardiography was performed at baseline and 4 weeks after MI (CX50, Philips, Netherlands). Parameters such as the left ventricular ejection fraction (LVEF), left ventricular end-diastolic dimension (LVEDD), left ventricular end-systolic dimension (LVESD) and fraction shortening (FS) were recorded. LVEF was measured using X-plane imaging and was calculated as follows: (LVVmax- LVVmin)/LVVmax. Three consecutive cardiac cycles were observed, and the average values were recorded as the final cardiac parameters.

### Detection of haemodynamics

Haemodynamic parameters were detected at baseline and at 4 weeks post-MI. The right femoral artery was punctured, and a 6 F pigtail catheter was used to perform LV angiography. The heart rate (HR), left ventricular systolic pressure (LVSP), and left ventricular end-diastolic pressure (LVEDP) of each animal were measured using the Mac-Lab system.

### Enzyme-linked immunosorbent assay (ELISA)

The myocardial expression of AngII and Ang(1–7) were detected using a commercial ELISA kit (Huamei Biological Co., Wuhan, China). The reaction system and standard curves were established according to the kit’s instructions.

### Real-time reverse transcription polymerase chain reaction (RT-PCR)

We used RT-PCR analysis to assay the mRNA expression of angiotensin-converting enzyme 2 (ACE2) and MasR in the LV. Total RNA extraction was performed according to the instructions provided with the Trizol reagent (CWbio. Co. Ltd, Cat# CW0581). Template cDNA was prepared from total RNA using the HiFi-MMLV cDNA reverse transcription kit (CWbio. Co. Ltd, Cat# CW0744). Quantitative PCR (Q-PCR), using an ABI7500 (Applied Biosystems, United States), was performed in triplicate for the amplification of target genes, while GAPDH was selected as an endogenous control. Each sample was run in duplicate with the following thermocycler protocol: an initial step 95 °C for 10 min, followed by 45 cycles at 95 °C for 15 s and 60 °C for 30 s. The PCR mix contained 0.4 uL (10 μmol) of forward and reverse primers, 10 uL of 2× UltraSYBR Mixture (CWbio. Co. Ltd, Cat# CW 0956), 2 uL of template cDNA and RNase-free water to a final volume of 20 uL. Melting curve analysis was used to confirm the specificity and identify of the PCR products, and relative gene expression changes were measured using the delta-delta Ct method, where X = 2-ΔΔCt. The primer sequences used are listed below in Table 1.

**Table 1:** Specific primers for Q-PCR

Gene names	Primers	Product size (bp)
ACE2	Forward: 5′- TTCAGCACAGTGGATCATCA-3′	95
	Reverse: 5′- CAAGTAATAAGCACTCCTGA-3′	
MasR	Forward: 5′- TGAGCAACAAGCTGAAGTCC-3′	124
	Reverse: 5′- AGGCACCTCCAGTCACACAA-3′	
P47phox	Forward: 5′- TGATTGCTGACTACAGCCAC-3′	136
	Reverse: 5′- AGGCTCCTCTGGTCCATCAA-3′	
GAPDH	Forward: 5′- CGGGCCGTCTTCCCCTCCAT-3′	138
	Reverse: 5′- CGGCCAGCCACGTCCAGACG-3′	

### Immunohistochemistry analysis

Cardiac expression of TH was detected by immunohistochemistry. Myocardial tissues were fixed with formalin, and after embedding and dehydrating, the LV tissue was sliced into 4-um sections. The sections were treated for 10 min with 1% methyl alcohol in H_2_O_2_ followed by treatment with 0.01 M citrate buffer (pH 6.0) for 10 min in a microwave oven, and the samples were then washed with PBS after cooling to room temperature. The sections were incubated overnight at 4 °C with primary antibody and again with biotinylated secondary antibody, followed by the addition of streptavidin-conjugated horseradish peroxidase. Immunohistochemistry staining for TH was performed using the DAB staining system according to the manufacturer’s instructions. Then, the nuclei were counterstained with haematoxylin. Three sections from each group were chosen for TH detection, and the amount of TH was assessed in five randomly chosen high-power fields from each sample. The investigator was blinded to the specimen’s source. The raw data were converted to an immunohistochemical score (IHS) to show the immunoreactivity of TH-positive nerves using an image analysis-based system [7].

### Statistical analysis

Experimental data were analysed using SPSS version 20.00 software. Quantitative data are presented as the mean ± SD. Group comparisons were subjected to analysis of variance (ANOVA), followed by the Newman-Keuls multiple comparison test to identify significant differences between individual groups. *P* < 0.05 was considered statistically significant.

## Results

One dog died due to a malignant arrhythmia on the 1^st^ day in the MI group, and one dog died on the 3^rd^ day in the MI + RDN group. There were no deaths in the control group.

### Baseline parameters

Dogs in each group were assessed for LVEDD, LVESD, FS, LVEF, LVEDP, LVSP and HR before MI (shown in Table 2) and there were no significant baseline differences between the three groups.

**Table 2:** Baseline data for each group

	Control group (*n* = 6)	MI group (*n* = 6)	MI + RDN group (*n* = 6)
Weight (kg)	16.00 ± 1.27	16.67 ± 1.51	16.50 ± 1.05
LVEDD (cm)	3.01 ± 0.15	3.15 ± 0.14	3.07 ± 0.17
LVESD (cm)	1.99 ± 0.16	2.11 ± 0.12	2.12 ± 0.13
FS (%)	33.81 ± 3.76	33.12 ± 1.52	31.00 ± 3.37
LVEF (%)	55.80 ± 2.99	58.60 ± 3.72	58.05 ± 3.00
LVEDP (mmHg)	6.17 ± 2.79	5.33 ± 2.07	5.17 ± 1.60
LVSP (mmHg)	124.17 ± 7.06	123.67 ± 12.31	123.33 ± 9.99
HR (bpm)	78.33 ± 10.99	81.33 ± 18.11	76.33 ± 21.95

Baseline data are presented as the mean ± SD with no significant differences between groups (all *P* > 0.05)

### Cardiac function and haemodynamic parameters at 4 weeks post-MI

At 4 weeks post-MI, compared to the control group, dogs from the MI and MI + RDN groups showed increased LVEDD, LVESD and LVEDP, while the LVEF, FS and LVSP values were all reduced. Importantly, compared with the MI group, parameters such as LVEF, LVEDD, LVESD and LVEDP were significantly improved in the MI + RDN group, but no significant difference was observed in FS and LVSP (*P* = 0.092 and *P* = 0.931, respectively). Additionally, there were no significant differences in HR among the three groups (shown in Table 3).

**Table 3:** Data for cardiac function and haemodynamic parameters at 4 weeks post-MI

	Control group (*n* = 6)	MI group (*n* = 5)	MI + RDN group (*n* = 5)
LVEDD (cm)	2.97 ± 0.15	3.75 ± 0.28^*^	3.40 ± 0.27^*#^
LVESD (cm)	1.96 ± 0.15	3.03 ± 0.16^*^	2.56 ± 0.16^*#^
FS (%)	34.03 ± 3.59	18.97 ± 6.32^*^	24.46 ± 4.26^*^
LVEF (%)	55.37 ± 2.98	37.04 ± 3.05^*^	41.72 ± 2.91^*#^
LVEDP (mmHg)	4.67 ± 1.63	16.00 ± 4.69^*#^	10.60 ± 3.05^*#^
LVSP (mmHg)	126.33 ± 13.29	104.60 ± 13.97^*^	103.80 ± 15.59^*^
HR (bpm)	80.50 ± 11.54	91.80 ± 32.95	74.40 ± 36.28

Values are presented as the mean ± SD

*P < 0.05 vs. control group; ^#^ *P* < 0.05 vs. MI group

### Cardiac oxidative stress levels

**Fig. 1:**
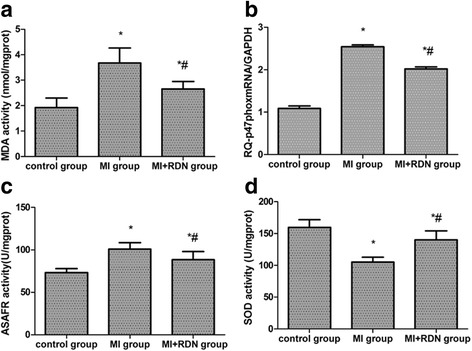
Cardiac oxidative stress levels in each group. **a** MDA, (**b**) p47^phox^ subunit, (**c**) ASAFR, (**d**) SOD. * *P* < 0.05 vs. control group;# *P* < 0.05 vs. MI group

As shown in Fig. 1, the cardiac infarct border zone was obtained to assess oxidative stress levels. At 4 weeks post-MI, the activities of MDA, p47^phox^, and ASAFR were all increased compared to the control group, while RDN treatment significantly reduced these changes (shown in Fig. 1a-c). SOD activity in the control, MI, and MI + RDN groups was 159.77 ± 11.90 U/mg prot, 105.14 ± 7.66 U/mg prot and 140.01 ± 14.10 U/mg prot, respectively (shown in Fig. 1d).

### Activity of the cardiac RAS

**Fig. 2:**
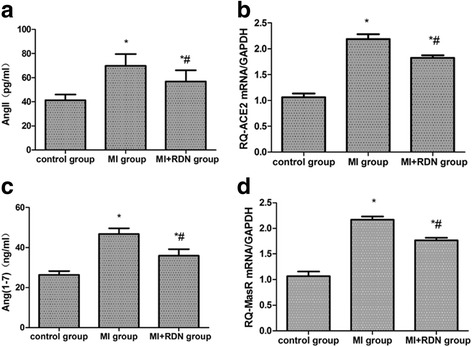
Detection of cardiac RAS activity. **a** AngII, (**b**) ACE2, (**c**) Ang(1–7), (**d**) MasR. The levels of AngII and Ang(1–7) were measured by ELISA. The mRNA levels of ACE2 and MasR were detected through RT-PCR, and GAPDH was used as an internal control. * *P* < 0.05 vs. control group; # *P* < 0.05 vs. MI group

Compared with the control group, the expression of AngII, ACE2, Ang(1–7) and MasR in myocardial tissue was significantly increased in the MI and MI + RDN groups. After intervention with RDN, the levels of AngII, ACE2, Ang(1–7) and MasR were significantly decreased in the RDN-treated group compared to the MI group (shown in Fig. 2a-d).

### Activity of the cardiac SNS

**Fig. 3:**
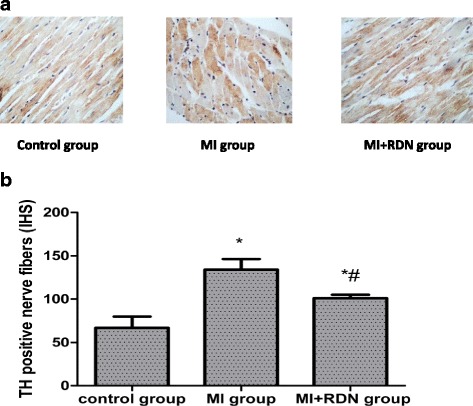
**a** TH immunohistochemical staining at the infarct border zone of nerve fibres (×400). Compared with the control group, the TH nerve fibres in the MI and MI + RDN groups were increased. However, after treatment with RDN, the quantity of TH nerve fibres was significantly decreased, and an orderly appearance of fibres was observed. **b** Expression of TH-positive nerves in cardiac tissues. * *P* < 0.05 vs. control group;# *P* < 0.05 vs. MI group

Four weeks post-MI, the relative expression of TH-positive nerves in the myocardial tissue from the control, MI and MI + RDN groups was 66.82 ± 13.09, 134.16 ± 12.06, and 101.14 ± 3.91, respectively. Compared with the control group, the amount of TH-positive nerves was markedly elevated in the MI and MI + RDN groups. Importantly, a decrease in TH-positive nerves was seen in the MI + RDN group compared with the MI group (shown in Fig. 3a-b).

### Correlation analysis

The expression of TH, AngII and Ang(1–7) was positively correlated with MDA (all *P* < 0.05) and negatively correlated with SOD (all *P* < 0.05). The correlation indexes are listed in Table 4.

**Table 4:** Correlation analysis

	TH (IHS)	AngII (pg/mL)	Ang(1–7) (pg/mL)
MDA (nmol/mg prot)	0.900	0.876	0.832
SOD (nmol/mg prot)	−0.818	−0.674	−0.806

### TH-positive nerves in renal tissue

**Fig. 4:**
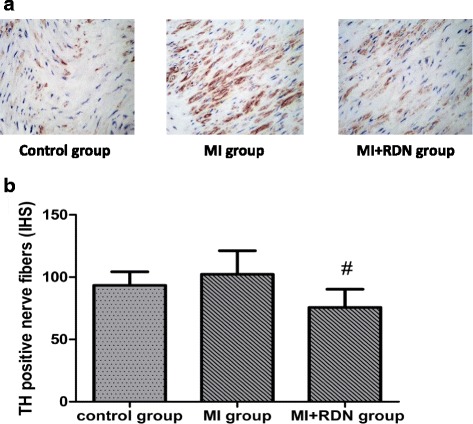
**a** TH immunohistochemical staining of nerve fibres in renal tissue (×400). Compared with the MI group, the density of TH nerve fibres was decreased in the MI + RDN group; no difference was observed between the control and MI + RDN groups. **b** Expression of TH-positive nerves in renal tissues. # *P* < 0.05 vs. MI group

Four weeks after MI, the relative expression of TH-positive nerves in renal tissue in the control, MI and MI + RDN groups was 93.44 ± 10.86, 102.26 ± 18.98, 75.69 ± 14.67, respectively. A decrease in TH-positive nerves was seen in the MI + RDN group compared to the MI group, while no significant difference (*P* = 0.071) was observed between the control and MI + RDN groups (shown in Fig. 4a-b).

### Comparison of serum creatinine at 4 weeks post-MI in each group

**Fig. 5:**
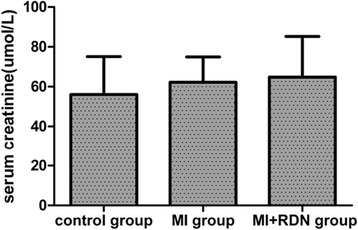
Serum creatinine levels at 4 weeks post-MI in each group. No differences were observed in serum creatinine among the three groups

The serum creatinine levels in the control, MI and MI + RDN groups were 56.00 ± 19.12 μmol/L, 62.20 ± 12.79 μmol/L and 64.80 ± 20.40 μmol/L, respectively. No difference in serum creatinine among the three groups was detected (*P* = 0.706, shown in Fig. 5).

## Discussion

Mounting evidence suggests that RDN is beneficial at improving cardiac function in both experimental animals and clinical patients. Hu et al. found that RDN was superior to monotherapy with a beta blocker, ACEI or ARB in decreasing plasma norepinephrine (NE) levels and improving cardiac function in rats with MI [8]. A significant improvement in diastolic function and a lowered LV mass index were also seen in RDN-treated refractory hypertension patients [9]. The present study showed that cardiac function was deteriorated after MI and that RDN inhibited the process of ventricular remodelling and improved cardiac function. Acute MI results in an increase in SNS and RAS activity in both the whole body and local myocardial tissues, which is proportional to the severity of post-MI HF [10, 11]. Additionally, reactive oxygen species (ROS), as the main effectors of oxidative stress, play an important role in the ventricular remodelling that occurs after MI [12]. Many studies have shown that RDN is effective at reducing the whole-body activity of the SNS and RAS. In our study, we explored the effect of RDN on cardiac oxidative stress and local activity of the SNS and RAS.

Previous studies have demonstrated that antioxidant therapy distinctly inhibits oxidative stress, delays ventricular remodelling and improves cardiac function after MI [13, 14]. Oxidative stress results from imbalance of the antioxidant and oxidant systems, which leads to excessive ROS production. The biological effects of nicotinamide adenine dinucleotide phosphate (NADPH) oxidase, one of the principle sources of ROS in the cardiovascular system [15], are regulated by antioxidant enzymes such as SOD and catalase. The NADPH p47^phox^subunit, composed of 390 amino acids, acts as a connector between the components of the membrane and cytoplasm and is essential for NADPH oxidase activation [16] and interstitial fibrosis [17]. MDA, the lipid peroxidate product, is used as a measure of the body’s oxidative levels. SOD is a vital antioxidant enzyme and effectively clears free oxygen radicals. In our study, we found that RDN reduced the high level of oxidative stress after MI. In addition, the activity of ASAFR in dogs from the MI + RDN group was lower than in those from the MI group. Regarding this phenomenon, we propose that RDN reduces the myocardial oxidative level mainly through decreasing the generation of superoxide rather than increasing the activity of ASAFR, which clears superoxide production.

It is well known that chronic sustained sympathetic over-activity results in the aggravation and deterioration of HF. Previously published studies have shown that RDN can reduce HR, NE overflow and muscle SNS activity [18–20]; meanwhile, RDN has also demonstrated beneficial effects in various conditions with high SNS activity, such as resistant hypertension, HF, malignant arrhythmia, impaired glucose tolerance and chronic renal dysfunction [1, 21–23]. Research has further demonstrated that TH-positive nerves can be used as an indirect indicator of sympathetic activity [24]. In our research, the effectiveness of removing sympathetic nerve activity by RDN was demonstrated by the decrease in TH-positive fibres in renal tissues. Moreover, the relative expression of TH nerve fibres, which reflects the local activity of the SNS, was also down-regulated in cardiac tissue, suggesting that RDN lowered the over-activation of the SNS in myocardial tissues.

The RAS is also a crucial mediator of myocardial fibrosis, pathological hypertrophy and HF [25, 26]. The adverse effects of the RAS on cardiovascular disease are mainly due to increased AngII, which exerts its effects through the classical ACE-AngII-AT1R axis. Importantly, the ACE2-Ang(1–7)-MasR axis, known as an endogenous counter-regulator of the RAS, has been confirmed to be protective in CHF [27]. The development of cardiovascular disease has been found to involve to both axes of the RAS [28]. ACE2’s major biological function is converting AngII into Ang(1–7), which is the physiological antagonist of AngII [29–31]. Ang(1–7) exerts vasodilatory, anti-proliferative, anti-inflammatory, and protective effects through activation of MasR. Previous studies have shown that ACE2 is increased in MI or HF [32–35]. As reported previously, the ACE2 gene was upregulated in HF patients and was associated with the degree of LVEDD and LVEF loss [36]. In agreement with previous investigations, our study showed that AngII and the compensatory ACE2-Ang(1–7)-MasR axis were increased in MI dogs, although these changes were reversed by ablation of renal sympathetic nerves. The exact mechanisms for the decreased expression of ACE2-Ang(1–7)-MasR after RDN are not fully understood; we speculate that RDN effectively inhibits LV dilatation and modifies cardiac function by reducing the activation of AngII to a significantly higher degree, which in turn leads to a relatively weakened compensatory mechanism. Importantly, a previous study reported that the enhanced ACE2 and Ang(1–7) levels were significantly reduced after treatment with Telmisartan in rats with HF [32], which is consistent with our results.

Cardiac SNS, RAS and oxidative stress mutually influence each other. AngII-induced phosphorylation of p47^phox^ [16] and myocardial hypertrophy induced by ROS mediate various signalling pathways [37]. Moreover, the AngII type 1 receptor (AT1R) blocker irbesartan and Ang(1–7) blocked NADPH oxidase activation and thus restored systolic function and carotid flow in an animal study [38, 39]. Other studies have shown that ROS increase SNS activity both in the central and peripheral nervous systems [40]. In our study, we found that RDN lowered the expression of AngII, MDA and TH-positive nerve fibres in the heart, and the change in MDA was positively correlated with AngII and TH. Our preliminarily hypothesis is that RDN improves cardiac function by reducing the activity of the cardiac AngII-ROS-SNS axis, which needs to be confirmed in future research.

Safety issues related to the procedure for RDN have attracted great attention. The REACH Pilot study evaluated the safety of RDN for HF, and no significant reduction in blood pressure or other haemodynamic disturbances were observed [1]. Mahfound et al. found that RDN reduced the renal resistance index without decreasing the glomerular filtration rate (GFR), and no renal artery stenosis or dissection was recorded [41]. Hering et al. demonstrated that RDN had no negative influence on patients with renal insufficiency [23]. In the present study, the LVSP and serum creatinine level showed no significant difference after RDN compared with the non-RDN treated group, thereby demonstrating the short-term safety of the RDN procedure.

## Conclusion

RDN exerts a protective effect against acute MI, which may be attributed to antioxidant effects and a decrease in the local activity of the SNS and RAS. In conclusion, RDN may serve as a new therapeutic treatment for MI patients by blocking these adverse mechanisms.

### Limitations

With regard to our study, some limitations should be considered. First, myocardial infarction was only validated by the pathological specimen and echocardiography, and we did not evaluate the infarct size, which reduces the persuasion to a certain extent. Second, the number of subjects was not large enough, which may have affected our statistical analysis. Third, due to lack of a control + RDN group, the effects of the intervention could not be excluded completely. Finally, the observation time was short. To confirm the validity of these results, studies both in humans and animals should be undertaken on a larger scale and with a longer observation time.

## Abbreviations

ACE2: Angiotensin converting enzyme 2
ACEI: Angiotensin-converting enzyme inhibitors
AMI: Acute myocardial infarction
Ang(1–7): Angiotensin 1 through 7
AngII: AngiotensinII
ARB: Angiotensin receptor blockers
ASAFR: Anti-superoxide anion free radical
AT1R: AngII type 1 receptor
CHF: Chronic heart failure
ELISA: Enzyme-linked immunosorbent assay
FS: Fraction shortening
GFR: Glomerular filtration rate
HR: Heart rate
IHS: Immunohistochemical score
LVEDD: Left ventricular end-diastolic dimension
LVEDP: Left ventricular end-diastolic pressure
LVEF: Left ventricular ejection fraction
LVESD: Left ventricular end-systolic dimension
LVSP: Left ventricular systolic pressure
MasR: Mas receptor
MDA: Malondialdehyde
NADPH: Adenine dinucleotide phosphate
NE: Norepinephrine
Q-PCR: Quantitative PCR
RAS: Renin-angiotensin system
RDN: Renal sympathetic denervation
RF: Radio frequency
RT-PCR: Real-time reverse transcription polymerase chain reaction
SNS: Sympathetic nervous system
SOD: Superoxide dismutase
TH: Tyrosine hydroxylase
